# Verification Study of Residual Activity Measurements After Yttrium-90 Radioembolization with Glass Microspheres

**DOI:** 10.1007/s00270-020-02504-7

**Published:** 2020-05-20

**Authors:** S. C. Ebbers, B. Kunnen, B. J. van Nierop, J. L. M. Bemelmans, G. C. Krijger, M. G. E. H. Lam, A. J. A. T. Braat

**Affiliations:** Department of Radiology and Nuclear Medicine, University Medical Center Utrecht, Heidelberglaan 100, 3508 GA Utrecht, The Netherlands

**Keywords:** Yttrium-90 microsphere, Radioembolization, SIRT, TheraSphere, Residual activity, Survey meter

## Abstract

**Objective:**

After yttrium-90 (^90^Y) radioembolization, residual activity and its consequences for dosimetric calculations are often not reported. The manufacturer for glass microspheres prescribes standard residual activity measurements by a survey meter, but the validity lacks evidence. This study aims to verify the accuracy of the survey meter approach for measuring residual activity of glass microspheres after treatment with glass microspheres.

**Methods:**

To validate the accuracy of the survey meter approach, the measured residual activity of glass microspheres by survey meter was compared with measurements by PET. A sample of these waste containers was also measured by dose calibrator to confirm the accuracy of the PET.

**Results:**

Twenty-four waste containers from glass microsphere treatments were prospectively scanned with ^90^Y-PET/CT. Bland–Altman plots showed substantial disagreement in residual activity measured by survey meter versus the residual activity measured by PET and dose calibrator, whereas the correlation between PET and dose calibrator was excellent (*ρ* = 0.99).

**Conclusion:**

This study found a significant disagreement between the residual activities measured by the survey meter, compared to measurements by PET and dose calibrator. If relatively high amounts of residual activity are encountered using the exposure rate measurement with a survey meter, additional quantification should be considered using either PET/CT or a dose calibrator measurement.

## Introduction

Selective internal radiotherapy (SIRT), also known as radioembolization, has proved to be an effective and safe treatment for various primary and secondary liver tumors [1–5]. Yttrium-90 (^90^Y) is the most commonly used isotope and its decay is mainly β^−^radiation, but 32 out of every one million decays are accompanied by positron emission, allowing PET-based quantification [6–8]. Accurate dosimetry is important, as several studies suggest a dose–response relationship [9–14]. However, there is little reported on the amount of residual activity (RA) in the administration system after administration and its consequences on treatment dosimetry [15]. For glass microspheres (TheraSphere®, Biocompatibles UK Ltd), the method of measuring RA recommended by the manufacturer is by exposure rate measurements (ERM) [16]. This method may be inaccurate, due to the differing geometry of the waste material. Two other widely available methods of measuring the RA are ^90^Y-PET/CT or a dose calibrator. Both methods are less likely to be influenced by the geometry of the material and thus expected to be more accurate. This study aims to verify the best quantitative method for measuring RA.

## Methods

### Procedures et al. [17]

Pre-treatment imaging and SIRT were performed using the procedures as previously described by Padia et al. [17]. Therapeutic activity was calculated according to the commonly used medical internal radiation dose (MIRD) method. After administration of the microspheres, the microcatheter, tubing, disposable surgical towel, gloves and V-vial (= vial containing therapeutic activity) were stored in one waste container for each injection. From August until October 2017, all waste containers from glass microsphere treatments were collected. All RA measurements were corrected for background activity and decay corrected to the date and time of microsphere administration.

### Survey Meter Method

**Fig. 1:**
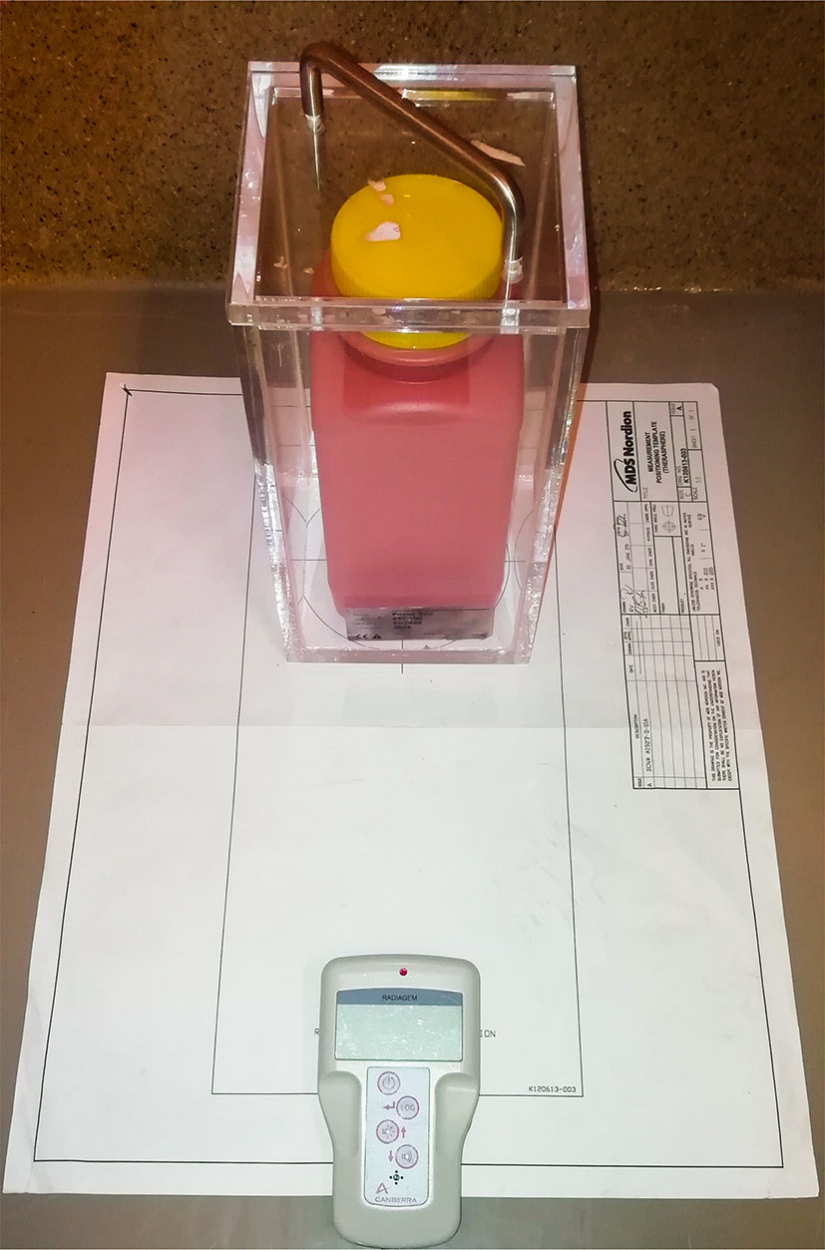
Exposure rate of each waste container was measured with a dose rate and survey meter (Radiagem™ 2000, Canberra). The fraction of residual activity was based on the mean exposure rate of the V-vial before treatment and the waste container after treatment, measured three times from all four sides. This fraction was multiplied by the calibrated activity provided by the supplier, resulting in the residual activity. No perspex shielding was used; however, to correct for the absence of perspex shielding a representable sample of waste containers was measured with and without shielding. Using simple linear regression, a correction factor was applied to all measurements

ERM and RA calculation were performed using the calibration sheet and calculation method provided by the manufacturer (Fig. 1). In addition to the manufacturer’s instruction, the exposure rates were measured three times per side, being the 12, 3, 6 and 9 o’clock sides, giving a total of twelve ERM per item and a more precise mean ERM.

#### ^90^Y-PET/CT

To confirm the accuracy of PET/CT, two unused vials of glass microspheres with a known calibrated activity (14 and 8 GBq) were scanned at multiple time points until decay had dropped below 5 MBq. All waste containers were scanned with PET/CT (Siemens Biograph mCT time of flight system; Siemens Healthcare, Erlangen, Germany) within 36 h after treatment. Acquisition included 10 min per PET position with approx. 43% overlap for entire field of view. PET images were reconstructed using 4 iterations with 21 subsets, a 5 mm full width at half maximum Gaussian post-reconstruction filter and reconstructed voxel size of 4.1 × 4.1 × 3.0 mm^3^. A co-registered CT scan was made for attenuation correction and to visualize the location of RA within the waste container. The waste container and V-vial were manually segmented, and activity recovery in each segmentation was calculated (ImageJ). By subtracting the V-vial segmentation from the entire waste container, the RA in the tubing system could be calculated.

#### Dose Calibrator

In a subset, waste material was measured using the dose calibrator (VDC-404, Veenstra Instruments, The Netherlands) within 48 h after treatment. Waste material was introduced into the dose calibrator in two separate parts, so that it would be sufficiently surrounded by the perspex shielding of the dose calibrator.

#### Data Analysis

All data analyses were performed using R version 3.5.2. The validity of ERM was measured by performing a two-sided paired samples t-test on the ERM and the PET measurements. A subset was also measured by dose calibrator, for which additional t-tests were performed. To better understand the agreement between the three methods of measurement, Bland–Altman plots were used [18]. Correlation of findings was performed using Spearman's rank correlation coefficient. Findings were deemed statistically significant with a *p*-value < 0.05.

## Results

Activity measurement of the two unused vials of glass microspheres by PET/CT correlated with calculated physical decay as long as the scanned activity was larger than 15 MBq. In total, twenty-four waste containers were scanned with PET/CT after the ERM. Twelve waste containers were also measured in the dose calibrator, after ERM and PET/CT.

**Fig. 2:**
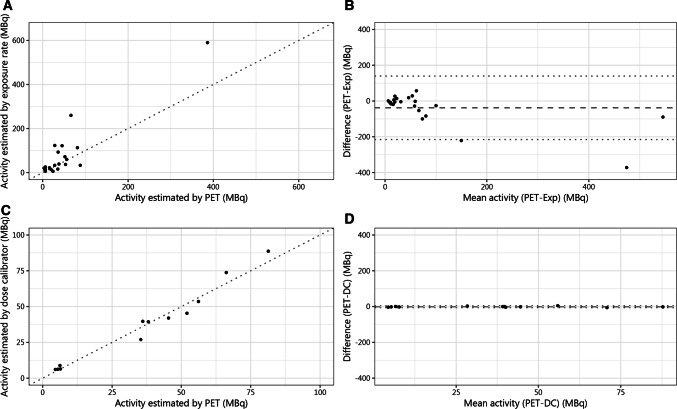
**A** correlation between residual activity measured by PET and exposure rate in MBq (megabecquerel). **B** Bland–Altman plot showing the level of agreement between measurements by PET and survey meter. **C** correlation between measurement of residual activities by PET and dose calibrator. **D** Bland–Altman plot showing level of agreement between measurements with PET and dose calibrator

Of the 24 waste containers, the median calibrated activity before administration was 1933 MBq (range [189; 10,100]). The median ERM was 0.61 µSv/h (IQR [0.36; 1.91]). The estimated median RA was 33 MBq (IQR [18; 98]) based on ERM and 32 MBq (IQR [6; 54]) based on PET/CT. According to the Bland–Altman plots, there was substantial disagreement between the measurements (Fig. 2) and rather low correlation (*ρ* = 0.76, *p* < 0.001). The paired samples t-test showed a significant mean difference between both measurements of 41 MBq (95% CI [5; 77], *p* < 0.05).

The correlation between the 12 measurements of PET and dose calibrator was much higher (*ρ* = 0.99, *p* < 0.001). The mean difference based on PET (median = 37 MBq; IQR [6; 53]) did not differ from the dose calibrator (median = 40 MBq; IQR [8; 47]), p = 0.850.

**Fig. 3:**
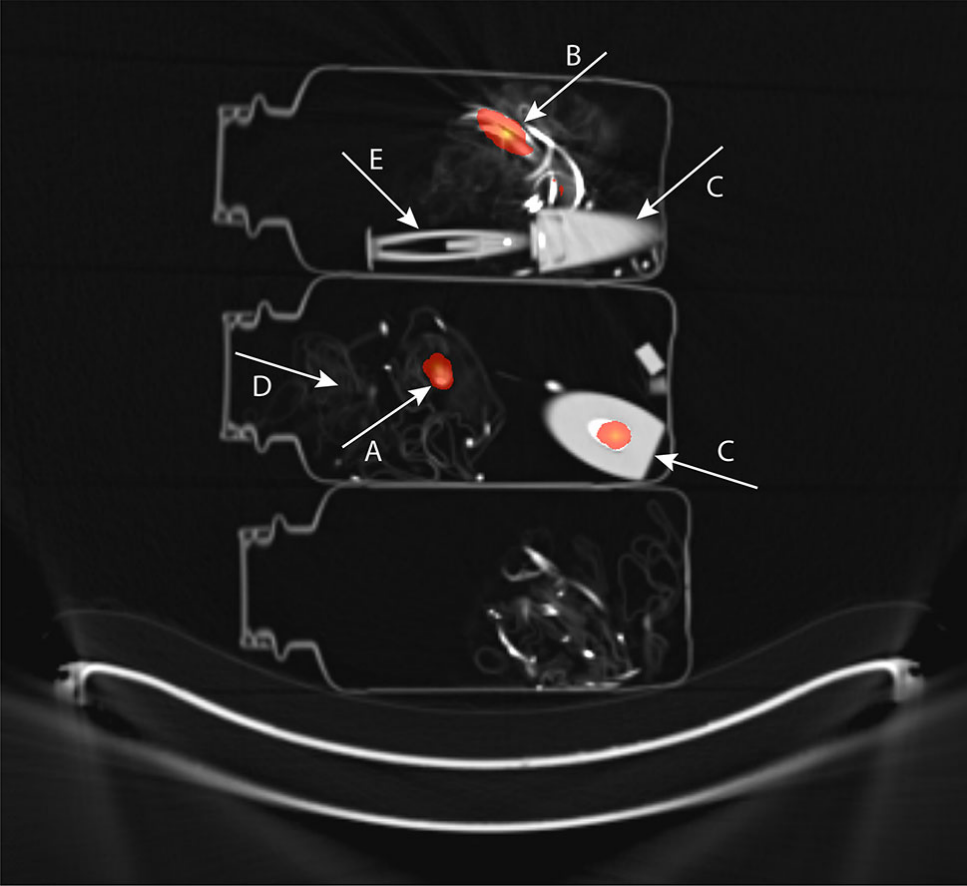
Combined images of PET and CT of three of the waste containers after radioembolization with glass microspheres showing that residual activity was found in the microcatheter connector (**A**), tubing (**B**) and V-vial (**C**). Other items that can be distinguished are the surgical cloth (**D**) and the plunger (**E**). ^89^Zr was used as an alternative isotope for acquisition and reconstruction (as ^90^Y as acquisition isotope was not available at that time). Activity recovery was corrected for the different half-life and positron branching ratio between ^89^Zr and ^90^Y

90% of RA was located outside the V-vial, and 10% was located inside the V-vial. Visually, most of the RA was located in the connector between tubing and microcatheter (Fig. 3). Only in one waste container, RA in the V-vial was > 1%.

## Discussion

This study shows that ERM of RA by survey meter is inaccurate and generally results an overestimation, especially in case of larger amounts of RA, as the absolute measurement error becomes more significant. Additionally, RA is mainly located in the connector of the microcatheter and not in the V-vial itself (Fig. 3).

The primary goal of measuring RA is to check whether a complete infusion was accomplished. Since ^90^Y is a pure beta emitter, the dose rate measurement relies on the measurement of bremsstrahlung, which is highly influenced by differences in geometry. The resulting inaccuracies do slightly impair the detection of small amounts of RA. However, when higher exposure rates are measured, a better estimate of the RA can be obtained by PET/CT or dose calibrator measurements.

A limitation of this study is that not all waste containers were also measured in the dose calibrator. This approach was chosen to limit potential radiation exposure to laboratory workers.

Studies on radioembolization tend to publish values of pre-treatment calculated or calibrated activities, without correcting for RA [19]. A more evidence-based and standardized approach for RA measurement and correction are needed for comparability in the literature. Each method of measurement has advantages and disadvantages (Table 1).

**Table 1:** Overview of advantages and disadvantages of measurements of residual activity after ^90^Y radioembolization

Method	Advantages	Disadvantages
Survey meter	Little radiation exposure and low costs	Inaccurate in case of high residual activities
Dose calibrator	Most accurate measurement and low costs	High risk of contamination and additional radiation exposure to laboratory workers
PET/CT	Very accurate measurement and little radiation exposure	Not available in all centers or costly

Based on our results, we recommend the use of PET or dose calibrator measurements to calculate and report RA in patients treated with SIRT, for whom RA > 50 MBq was found by exposure rate measurements. As an alternative, multiple studies demonstrated the usefulness of calculating the total absorbed dose based on the post-treatment PET/CT scans; however, this might be time-consuming and acquiring post-treatment ^90^Y-PET-CT might not be local clinical practice [20–22]. Finally, because of the retention of glass microspheres in the connector of the microcatheter, we emphasize the importance of thorough flushing and, after administration, the microcatheter should not be disconnected upon disposal to avoid contaminating the angiography suite.

## Conclusion

This study found a significant disagreement between the residual activities measured by the survey meter, compared to measurements by PET and dose calibrator. If relatively high amounts of residual activity are encountered using the exposure rate measurement with a survey meter, additional quantification by PET/CT or dose calibrator should be considered.
